# Effects of speed, agility, and quickness (SAQ) training on soccer player performance—a systematic review and meta-analysis

**DOI:** 10.1371/journal.pone.0316846

**Published:** 2025-02-21

**Authors:** Min Sun, Kim Geok Soh, Shuzhen Ma, Xinzhi Wang, Junlong Zhang, Azhar Bin Yaacob

**Affiliations:** 1 Faculty of Education Studies, Universiti Putra Malaysia, UPM Serdang, Serdang, Selangor, Malaysia; 2 Department of Physical Education, Yuncheng University, Yuncheng, Shanxi Province, China; Universita degli Studi di Verona, ITALY

## Abstract

**Background:**

Previous studies have reported on the impact of Speed, Agility, and Quickness (SAQ) training on the performance of soccer players. However, there is still controversy regarding the results. This systematic review and meta-analysis aim to accurately assess the effects of SAQ training on the performance of soccer players.

**Methods:**

We conducted a comprehensive search on March 15, 2024, using Web of Science, PubMed, Scopus, and EBSCOhost. Eligibility criteria for selecting studies were established based on the PICOS framework: (i) Population—healthy soccer players; (ii) Intervention—SAQ training; (iii) Comparison condition (conventional training or traditional training); (iv) Outcome—physical performance (speed, agility, strength, etc.); (v) Study design—randomized controlled trials. The PEDro scale was employed to evaluate the methodological quality of each study, and a random-effects model was used for the meta-analysis.

**Results:**

A total of 11 studies met the inclusion criteria for the systematic literature review. One study with low PEDro score was excluded, and one was excluded based on Cochrane bias risk assessment. Finally, 9 studies were included in the meta-analysis, comprising 498 soccer players. Overall, the results indicated a significant impact of SAQ training on physical qualities and dribbling speed among soccer players. Specifically, there was a moderate effect size for sprint performance (5m, 10m, 20m) (ES = 0.75; p < 0.01), change of direction ability (COD) (ES = 0.35; p < 0.001), power (vertical and horizontal jumps) (ES = 0.67; p < 0.01), while flexibility showed no significant impact (ES = 0.11; p > 0.05). Moreover, change-of-direction dribbling demonstrated a significant effect (ES = 0.58; p < 0.01).

**Conclusion:**

Overall, SAQ training effectively enhances speed, COD, explosiveness, and change-of-direction dribbling specific performance in adolescent soccer players, particularly in sprinting. However, it does not have an advantage in improving flexibility. Further high-quality studies encompassing a broader range of exercises are needed to fully determine the effectiveness of SAQ training in improving other physical qualities and technical skills of soccer players, as well as ultimately enhancing match performance.

## Introduction

With the rapid growth in the demands of soccer matches, players must possess higher levels of physical fitness, technical skills, and tactical abilities [1]. Developing effective training methods to enhance athletes’ performances has been a subject of great interest [2]. Soccer is characterized by frequent changes in activity levels, alternating between high-intensity actions like sprinting, jumping, shooting, and acceleration or deceleration, and lower-intensity activities such as jogging, walking, and standing [3,4]. Among these, the ability to rapidly change speed and sprint is the most common ability associated with scoring goals in soccer [5]. For instance, the ability to swiftly dribble past opposing players into the opponent’s territory is often regarded as a genius-like performance [6], and such breakthroughs can provide significant tactical advantages to the team [7]. Some studies indicate that the characteristic of high-speed movements during matches involves constant changes in speed and direction, although they constitute less than 12% of the overall soccer performance; however, these actions significantly influence the outcome of the game [8,9]. These abilities are also considered important indicators for distinguishing between elite and non-elite players [5]. Therefore, effective training to enhance these abilities is crucial for optimizing players’ performance in matches.

As fundamental determinants of team sports performance, coaches and practitioners must enhance agility through appropriate training strategies [10,11]. Various training modalities have been adopted to improve speed and agility performance in soccer players, such as core strength training [12], plyometric training [13], SAQ training [14–16], and Small-Sided Games patterns [17]. SAQ (Speed, Agility, and Quickness) training is thought to improve soccer players’ reaction to stimuli, boost acceleration, enhance multi-directional movement, and facilitate rapid changes in direction or stopping, contributing to a faster, more efficient, and consistent performance [18]. Additionally, SAQ training encompasses speed, agility, and quickness through a series of soccer-specific exercises performed with optimal movement patterns, believed to optimize muscle recruitment, thereby conserving energy and time [16]. The accompanying neurophysiological adaptations are associated with enhanced efficiency around the stretch-shortening cycle (SSC) [19]. Moreover, the benefits of this approach may also encompass improvements in strength and skill [20,21].

While a considerable body of research has reported the potential benefits of SAQ training on physical and skill performance, conflicting and controversial results still exist regarding its effects on speed and agility [14,21,22]. Furthermore, despite the widespread application of SAQ across various sports to enhance neuromuscular functions [23–26], there is currently no systematic review or meta-analysis in the literature focusing on the impact of SAQ on the physical and skill performance of soccer players. Therefore, the main goal of this systematic review and meta-analysis was to examine existing research on how SAQ training influences soccer players’ performance, aiming to identify the best practices and improve comprehension of its effects on athletes. To achieve this, all experimental studies comparing SAQ training with control groups of soccer players were reviewed. All selected studies met the criteria for randomized controlled trials (RCTs).

## Methodology

### Protocol and registration

This systematic review and meta-analysis adhered to the PRISMA guidelines [27] (S3 Table), with the protocol registered on Inplasy.com.(INPLASY202430077)

**Systematic Review Registration: https://inplasy.com/inplasy-2024-3-0077/**.

### Eligibility criteria

The selection of relevant studies was conducted using the PICOS method (Table 1). The chosen studies had to be published in academic journals and written in English. Studies were deemed eligible if they discussed the physical fitness, cognitive, or skill performance of soccer players. To avoid confusion, studies focusing solely on agility, speed, or quickness without explicit SAQ interventions were excluded. Additionally, this review only considered randomized controlled trials.

**Table 1:** Eligibility criteria based on PICOS (participation, intervention, comparison, outcome, and study design).

PICOS	Criteria
Participation	Soccer players
Intervention	Speed, agility, and quickness, (SAQ) training
Comparison	SAQ vs Regular training
Outcome	Physical, cognitive, and skill performance
Study design	Randomized controlled trial

### Search strategy and selection process

On March 25, 2024, we conducted a comprehensive search across four electronic databases—Web of Science Core Collection, SPORTDiscus, PubMed, and SCOPUS—to find articles relevant to our topic. We utilized a combination of keywords and Boolean operators in our searches, employing terms such as "SAQ training" or "speed, agility, quickness training*" in conjunction with "speed," "flexibility," "agility," "athlete performance," "sports performance," or "physical performance," and "soccer player*," "football player*," "soccer athlete*," or "football athlete*." Additionally, we performed manual searches through Google Scholar for supplementary materials and reviewed the reference lists of all identified articles to find any relevant studies not captured in the initial database searches. The data collection process was further supported by experienced librarians to ensure accuracy and thoroughness (S1 Table).

**Fig 1:**
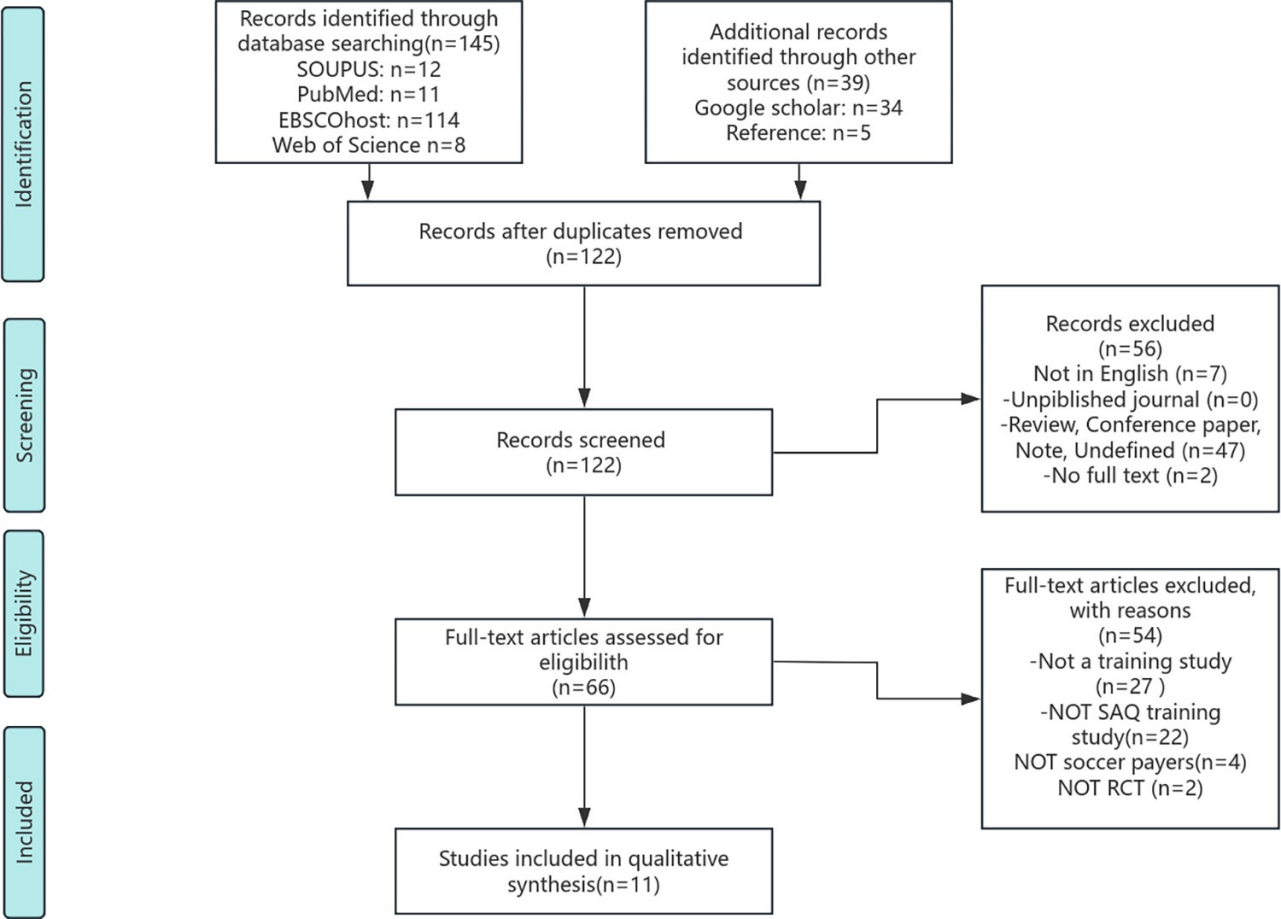
PRISMA flow diagram.

The study selection process comprised four pivotal stages (Fig 1). Initially, the removal of duplicate articles was executed. Subsequently, in the second stage, exclusion criteria were primarily directed toward articles exhibiting unclear relevance to the topic or those scripted in languages other than English. Moreover, conference abstracts, books, book chapters, and non-peer-reviewed journal papers were systematically excluded. The comprehensive screening process entailed a meticulous review of pre-established eligibility criteria. Furthermore, studies lacking training interventions or SAQ training interventions, as well as those not involving soccer players or not conducted as randomized controlled trials, were systematically excluded. This rigorous procedure was conducted independently by two reviewers (MS, SZM), with any disparities resolved through further discourse. Should the need arise, a third reviewer (KGS) was enlisted to provide assistance until a consensus was achieved (S4 Table).

### Data extraction

The data extracted from the included literature encompass the following details: (i) first author’s name and publication year; (ii) characteristics of participants, including age, gender, sample size, and athletic level; (iii) characteristics of SAQ training intervention, such as training duration, frequency, timing, and types of exercises; (iv) assessment of athlete performance, including physical attributes, cognitive performance, and skill performance; and (v) mean and standard deviation of outcomes for the SAQ group and control group. The detailed information for each study were recorded by two independent reviewers (MS, JLZ) using Microsoft Excel spreadsheets and validated for accuracy by a third reviewer (KGS) (S5 Table).

### Quality assessment and risk of bias

In this review, we used the PEDro scale and the Cochrane Risk of Bias tool for quality and risk of bias assessment. Several studies support the combined use of these two tools to provide a more comprehensive evaluation [28].

Two reviewers (MS, SZM) independently utilized the PEDro scale, which has been proven to be a reliable measure for assessing the methodological quality of systematic review methods [28]. The third author (XZW) verified the results, and all three reviewers reached an agreement. The recommended scores for this scale are as follows: Scores below 4 are categorized as ’poor,’ scores between 4 and 5 are labeled ’fair,’ scores from 6 to 8 are deemed ’good,’ and scores between 9 and 10 are rated as ’excellent. [28]. The PEDro scale includes 11 criteria to assess methodological quality. For each criterion met, one point is added to the PEDro score, which can range from 0 to 10. Standard 1, which pertains to external validity, was excluded from the assessment of study quality. Therefore, this review is based on ten studies and analyzes the impact of SAQ training on the performance of soccer players.

**Fig 2:**
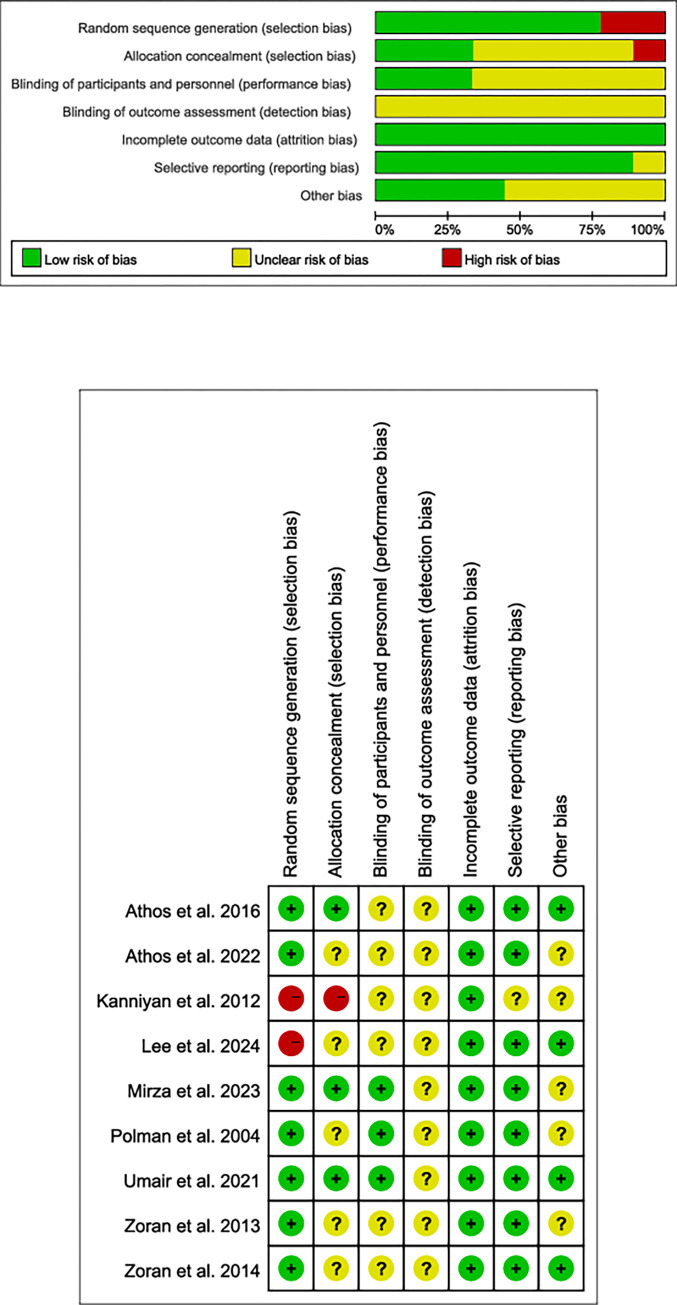
Risk of bais assessement.

Two additional reviewers (XZW, JLZ) assessed the risk of bias using Revman Manager 5.4.1, based on the Cochrane Collaboration guidelines. Each of the seven domains was assigned a rating of ’low risk of bias,’ ’unclear risk of bias,’ or ’high risk of bias.’ Subsequently, the overall risk of bias for each study was determined (Fig 2).

In summary, four independent reviewers utilized the PEDro and Revman tools. Consensus was reached between them or discrepancies were resolved by a third reviewer.

### Statistical analysis

While meta-analysis comparisons could be conducted with only two studies [29], several studies included in this research have small sample sizes [21,22]. Therefore, meta-analysis was performed only on data reporting performance outcomes from three or more studies. Effect sizes (ES) (Hedges’ g), means, and standard deviations of measures before and after intervention were computed using performance data, and intervention post-data were standardized using performance measures. In cases where data values are inaccessible, such as when they are omitted or presented graphically, contact the corresponding author of the study to obtain the necessary information.

Effect sizes in the meta-analysis were calculated using the mean and standard deviation of the outcome variables. An inverse variance random-effects model was applied to express the effect sizes as standardized mean differences (SMD) with corresponding 95% confidence intervals (CI). Interpretations of standardized mean differences were as follows: less than 0.2 was considered trivial; 0.2 to 0.6 was small; greater than 0.6 to 1.2 was moderate; more than 1.2 to 2.0 was large; over 2.0 to 4.0 was very large; and above 4.0 was deemed extremely large [30]. The control group was proportionally divided in studies with multiple intervention groups to facilitate comparison among all participants [31]. Statistical heterogeneity was measured using the *I*^2^ test (*I*^2^ ≤ 25% indicates low heterogeneity, 25% < *I*^2^ < 75% indicates moderate heterogeneity, *I*^2^ ≥ 75% indicates high heterogeneity) [32]. To assess publication bias risk, the extended Egger’s test was used [33], and sensitivity analysis was applied to cases with significant results from Egger’s test. The analyses were carried out using Comprehensive Meta-Analysis software (version 3; Biostat, Englewood, NJ, USA), with statistical significance defined as p < 0.05.

## Results

### Study selection

Initially, 145 papers were identified from database searches. Through Google Scholar and reference lists, 39 more studies were identified. After eliminating duplicate entries, 122 records were retained. Titles and abstracts of these records were then reviewed, leading to the selection of 66 studies for full-text evaluation. Following this, 54 studies were excluded after a thorough review (refer to Fig 1). Ultimately, 11 papers met the inclusion criteria, with 9 suitable for meta-analysis.

### Quality assessment and risk of bias

Table 2 presents the quality assessment of the included literature using the PEDro scoring tool. It is worth noting that one study was of lower quality and was not considered [34].

**Table 2:** Physiotherapy evidence database (PEDro) scale ratings.

Study name	N1	N2	N3	N4	N5	N6	N7	N8	N9	N10	N11	Total	Study quality
Mirza et al. 2023[14]	0	1	1	1	0	0	0	1	1	1	1	7	Good
Umair et al. 2021[15]	0	1	1	1	0	0	0	1	1	1	1	7	Good
Zoran et al. 2013[36]	1	1	0	1	0	0	0	1	1	1	1	7	Good
Polman et al. 2004[20]	0	1	0	1	0	0	0	1	1	1	1	6	Good
Zoran et al. 2014[37]	1	1	0	1	0	0	0	1	1	1	1	7	Good
Athos et al. 2016[11]	0	1	0	1	0	0	0	1	1	1	1	6	Good
Kanniyan et al. 2012[35]	0	0	0	1	0	0	0	1	1	1	1	5	Fair
Lee et al. 2024[21]	1	1	0	1	0	0	0	1	1	1	1	7	Good
Azmi et al. 2018[34]	0	0	0	0	0	0	0	0	1	1	1	3	Poor
Mario et al. 2011[16]	0	1	0	1	0	0	0	1	1	1	1	6	Good
Athos et al. 2022[22]	0	1	0	1	0	0	0	1	1	1	1	6	Good

Note: A detailed explanation for each PEDro scale item can be accessed at https://www.pedro.org.au/english/downloads/pedro-scale.

Regarding bias risk, the Bias Risk Tool in Revman 5.4.1 was utilized, and after deliberation among three authors, one study [35] was excluded due to high bias risk in randomization and allocation (Fig 2). Another study [21], although using incomplete jersey numbers for randomization, was still considered to pose a "high risk of bias." Insufficient information was available to assess the blinding of outcome assessments in all studies (S6 Table). Additionally, five studies [11,21,22,36,37] were classified as having an "unclear bias risk" in the randomization process due to unknown allocation concealment. Four studies did not explicitly specify other bias risks [14,20,22,36].

### Study characteristics

Table 3 presents the characteristics of participants and interventions in the randomized controlled trials (RCTs) included in this review [11,14,15,20–22,36–38]. A total of 498 soccer players were included (257 in the experimental group and 241 in the control group). Among them, 350 (70.3%) were male, 55 (11%) were female, and gender information was not reported for 93 (18.7%) participants. The age of the participants ranged from 8 to 12 years and from 18 to 25 years. Regarding the intervention, the experimental group underwent SAQ training, while the control group received regular training. The training duration in all included RCT studies ranged from 4 to 12 weeks. SAQ training sessions lasted from 20 to 170 minutes per session, with a frequency of 2 to 4 times per week. In terms of participants’ level of play, 4 studies selected national club soccer players, 3 studies categorized participants as pre-adolescent soccer players, one study was conducted in a soccer academy, and one study included university-level soccer players.

**Table 3:** Characteristics of participants and SAQ interventions in the included studies.

Study	N	Sex	Age	Level	Comparison	Intervention			Outcome
						SAQ Training	W/F/time	Intensity	
**Athos et al. 2022[22]**	**EG = 11** **CG = 10**	**NR**	**EG = 9.7±0.4** **CG = 9.5±0.6**	**prepubescent**	**EG = SAQ** **CG = SSG**	**Footwork exercises, sprinting, changing directions, and auditory and visual stimulation, etc.**	**4/2/25**	**75%-88%**	**SP5↑SP20→CODS90→** **Cognitive EG↑**
**Mirza et al. 2023[14]**	**EG = 18** **CG = 15**	**NR**	**8.59±0.69**	**prepubescent**	**EG = SAQ** **CG = RT**	**5-20m sprint with ball, 505 pole, etc**	**4/2/NR**	**As high as possible**	**SP5↑SP10↑SP20→** **505 CODS DL→90° turn with ball↑** **Slalom 10m with ball↑**
**Umair et al. 2021[15]**	**EG = 33** **CG = 33**	**Male**	**EG = 19.64±0.91** **CG = 18.57±0.50**	**Soccer School**	**EG = SAQ EQ** **CG = RT**	**Fast feet, sprint, reaction ball, rope ladder, resistance band, etc.**	**6/3/60**	**As high as possible**	**SP20↑Illinois CODS↑vertical jump↑**
**Kanniyan et al.[35]**	**EG = 10** **CG = 10**	**Male**	**18–26**	**college**	**EG = SAQ** **CG = RT**	**No details**	**6/3/60-75**	**NR**	**SP30↑ 400 meters↑ Shuttle run↑**
**Zoran et al.2013[36]**	**EG = 66** **CG = 66**	**Male**	**EG = U19** **CG = U19**	**national club**	**EG = SAQ** **CG = RT**	**Fast feet, jumping, sprinting, zigzag running, waiting for reaction**	**12/4/120-170**	**70%-92%**	**SP90trun↑SP90 with ball↑** **SP180 turn↑Slalom test with ball↑**
**Polman et al. 2004[20]**	**EG1 = 12** **EG2 = 12** **CG = 12**	**Female**	**21.2±3.1**	**national club**	**EG1 = SAQ** **EG2 = SAQ EQ** **CG = RT**	**Sprinting skills, reaction training, ladder training, jumping, strength training and speed endurance training**	**12/2/60**	**70%-95%**	**Aerobic capacity all↑** **SP25 EG1,EG2↑flexibility all↑** **Agility Left EG2›EG1›CG↑** **Agility Right EG1›EG2.CG**
**Zoran et al. 2014[37]**	**EG = 66** **CG = 66**	**Male**	**EG = 18.5±0.4** **CG = 18.6±0.6**	**national club**	**EG = SAQ** **CG = RT**	**Various running, jumping, agility ladder, reaction ball, sprinting, etc.**	**12/4/120**	**70%-90%**	**SP5↑SP10↑SP20→** **flexibility→**
**Athos et al. 2016[11]**	**EG = 20** **CG = 19**	**NR**	**EG = 10.5±0.30** **CG = 10.7±0.21**	**prepubescent**	**EG = SAQ** **CG = RT**	**Fast feet, sprints, rope ladders, visual stimulation, chase runs, mirror exercises, etc.**	**12/2/25**	**80%-100%**	**SP5↑SP20↑reactive agility↑ CODS→**
**Lee et al. 2024[21]**	**EG = 9** **CG = 10**	**Female**	**18.89±0.80**	**College**	**EG = SAQ** **CG = RT**	**Speed wall drills, COD, agility ladder drills, reaction balls, mirror games, etc.**	**8/3/40**	**80%-100%**	**SP5 SP10 CODS RA↑ with the ball↑** **SP5 SP10 RA: EG vs CG no significant difference** **SP20 SP30 with ball EG vs CG significant difference**

N, Number; EG, Experimental group; CG. Control group; NR, Not Reported; SAQ, speed agility, quickness; SSG, Small-Sided Games; RT, Regular training; W, week; F, Frequency; SP5, 5-meter sprint; SP10, 10-meter sprint; CODS, Change of Direction Speed; SJ, test values in squat jump; CMJ, countermovement jump; MAX, maximal CMJ; CJS, continuous jumps; NL, Non-dominant leg; DL, Dominant leg; RA, Reaction agility; ↑, increased significance; →, No significant improvement.

### Meta-analysis results

This meta-analysis focuses on 9 studies assessing the performance of socceplayers, specifically measuring sprint speed, agility, strength, flexibility, and dribbling agility. The data used for the meta-analysis are available in S2 Table.

**Fig 3:**
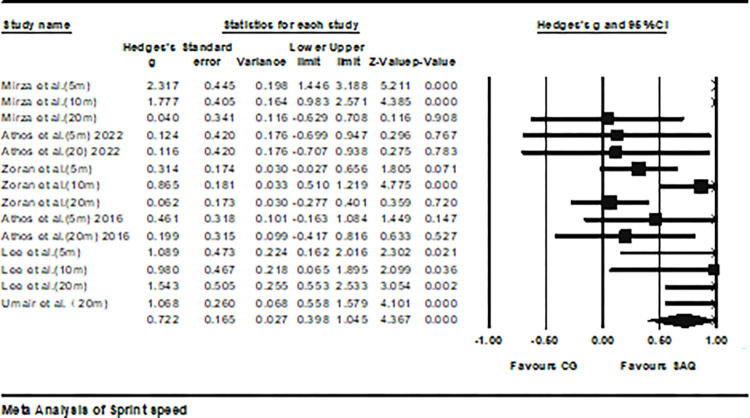
Depicts the forest plot illustrating the variation in sprint performance among athletes participating in SAQ training compared to the control group. The effect sizes (Hedges’ g) along with 95% confidence intervals (CI) are shown. The area of each square in the plot indicates the weight assigned to each study.

**Fig 4:**
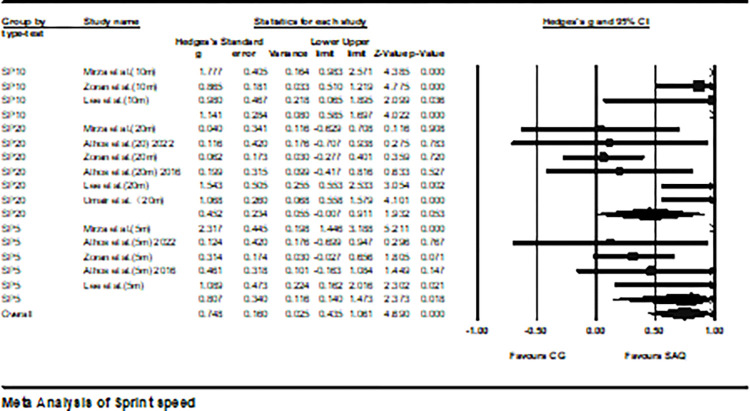
The forest plot shows the differences in sprint distance performance between athletes undergoing SAQ training and those in the control group. The plot depicts effect sizes (Hedges’ g) along with 95% confidence intervals (CI). The dimensions of the squares in the plot denote the weight of each study.

Six studies provided data on sprint speed, involving a total of 14 experimental and control groups (total n = 346). The results indicated a moderate effect of SAQ training on sprint speed (ES = 0.75; 95% CI = 0.44–1.06; P < 0.001; *I*^2^ = 75.6%; Egger’s test p = 0.11; Fig 3). In the analysis, the weights of each study ranged from 5.88% to 9.36%. Further analysis in Fig 4 revealed a moderate effect of SAQ on SP5 (ES = 0.81; 95% CI = 0.14–1.47; p < 0.05; *I*^2^ = 80%); a substantial effect on SP10 (ES = 1.41; 95% CI = 0.59–1.70; p < 0.01; *I*^2^ = 52.7%); and a slight effect on SP20 (ES = 0.45; 95% CI = -0.01–0.91; p = 0.05; *I*^2^ = 71.1%).

**Fig 5:**
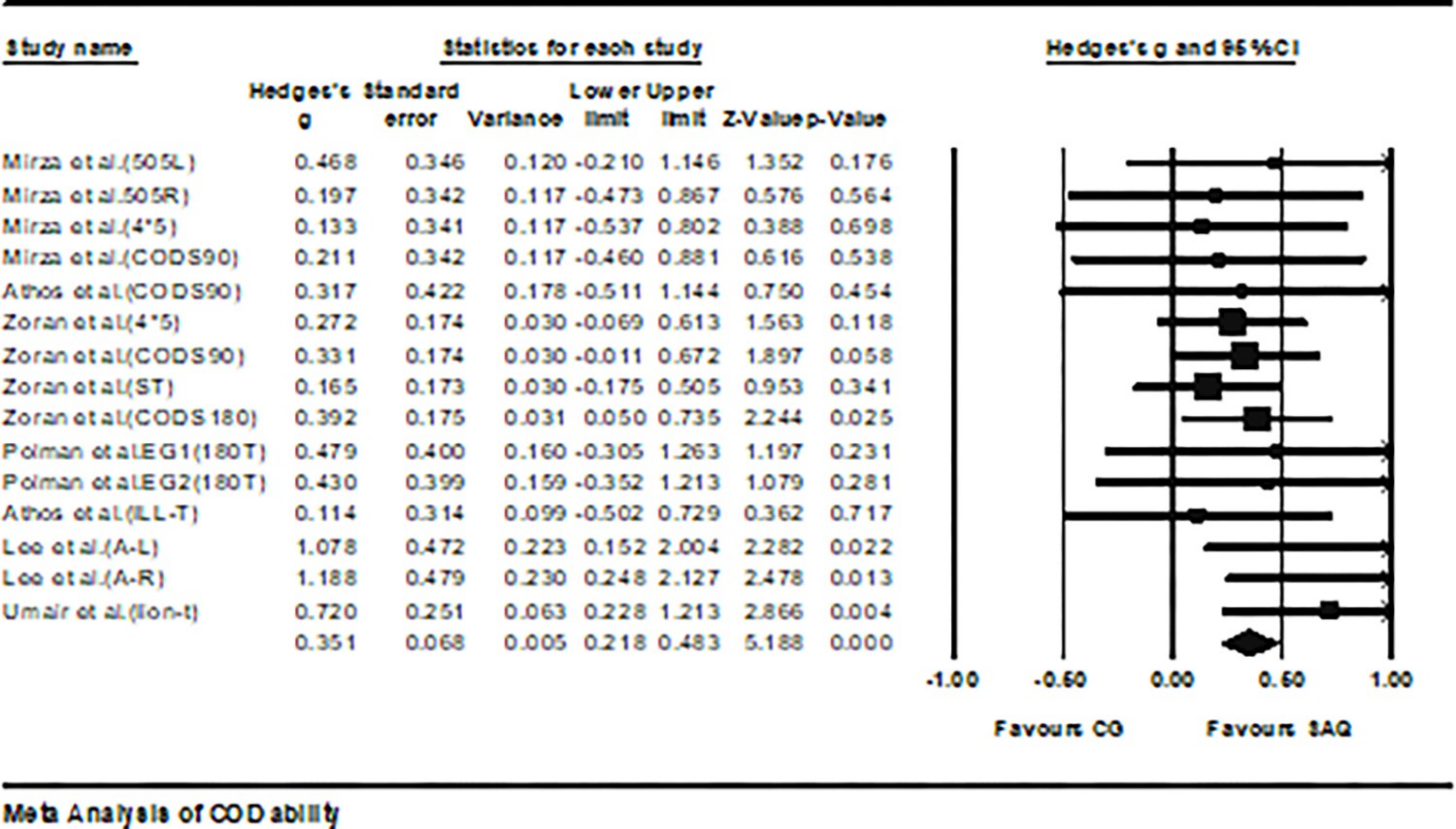
Forest plot illustrating the variation in COD performance among athletes participating in SAQ training compared to the control group. The values shown indicate effect sizes (Hedges’ g) along with 95% confidence intervals (CI). The area of each square in the plot corresponds to the weight of the study.

Seven studies provided data on agility performance, involving 14 experimental groups and 13 control groups (total n = 336). The impact of SAQ on change-of-direction ability (COD) performance in soccer players was small (ES = 0.35; 95% CI = 0.22–0.48; P < 0.01; *I*^2^ = 0.0%; Egger’s test, p = 0.13) (Fig 5). In the analysis, the weight range of each study ranged from 1.99% to 15.19%.

**Fig 6:**
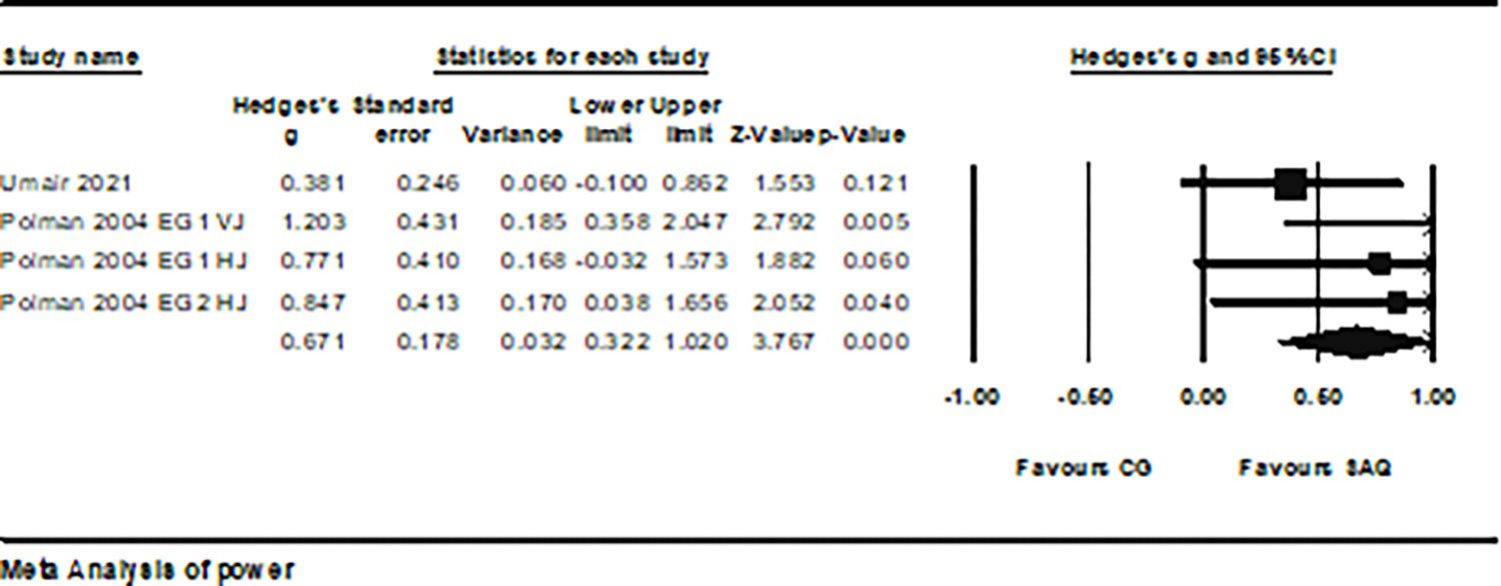
The forest plot illustrates the changes in power performance between athletes participating in SAQ training and the control group. The displayed values represent the effect size (Hedges’ g) with a 95% confidence interval (CI). The size of the square symbol reflects the statistical weight of the study.

Data on power were obtained from two studies, which included 5 groups in the experimental condition and 3 in the control condition (total n = 102). The Egger’s test indicated a p-value of 0.023, and following sensitivity analysis, one study was excluded [20], allowing Egger’s test p > 0.05. Therefore, the final consideration involved four experimental groups and three control groups (Egger’s test showed p = 0.07). SAQ had a moderate impact on the power performance of soccer players (ES = 0.67; 95% CI = 0.32–1.02; P < 0.001; *I*^2^ = 4.9%; Egger’s test, p = 0.068; Fig 6). In the analysis, the weight range of each study ranged from 17.93% to 47.36%.

**Fig 7:**
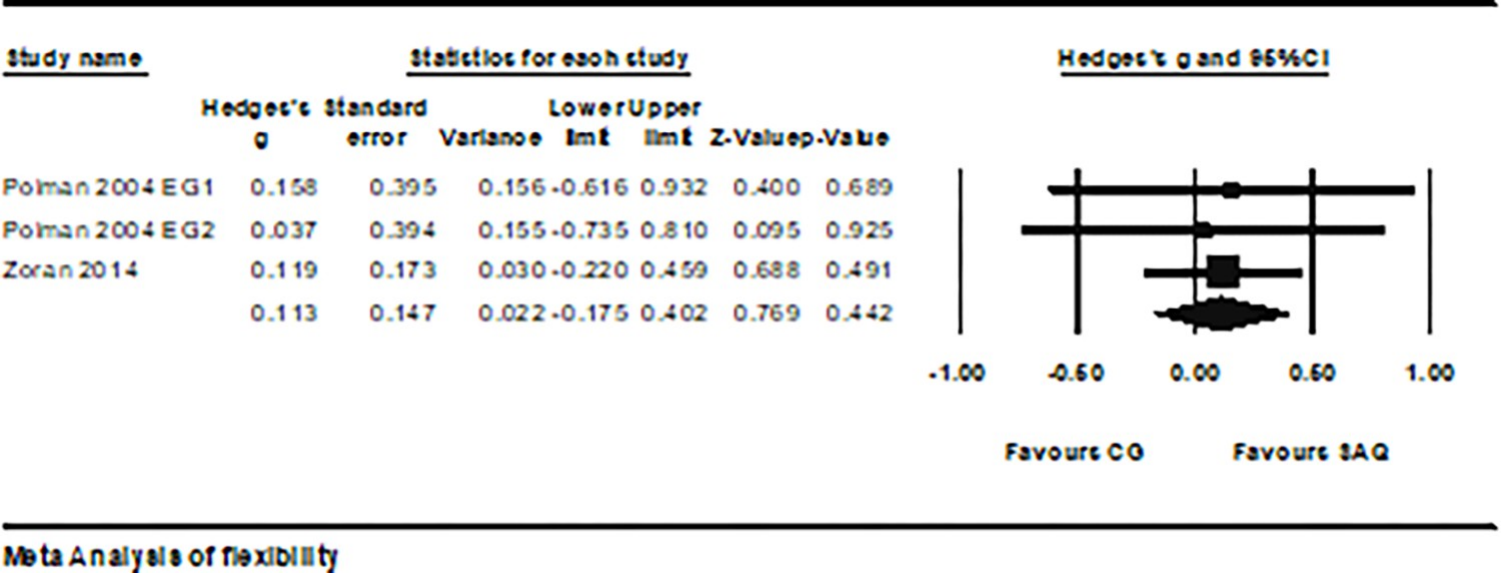
The forest plot illustrates the changes in flexibility performance between athletes participating in SAQ training and the control group. The effect sizes (Hedges’ g) and their corresponding 95% confidence intervals (CI) are illustrated. The dimensions of the square markers indicate the weight of each study.

2 studies provided data on the flexibility of athletes, involving 3 experimental groups and 2 control groups (total n = 168). SAQ training did not affect the flexibility of soccer players (ES = 0.11; 95% CI = -0.17–0.40; P > 0.05; *I*^2^ = 0.0%; Egger test, p = 0.81; Fig 7). The weight values of each study ranged from 13.89% to 72.17% in the analysis.

**Fig 8:**
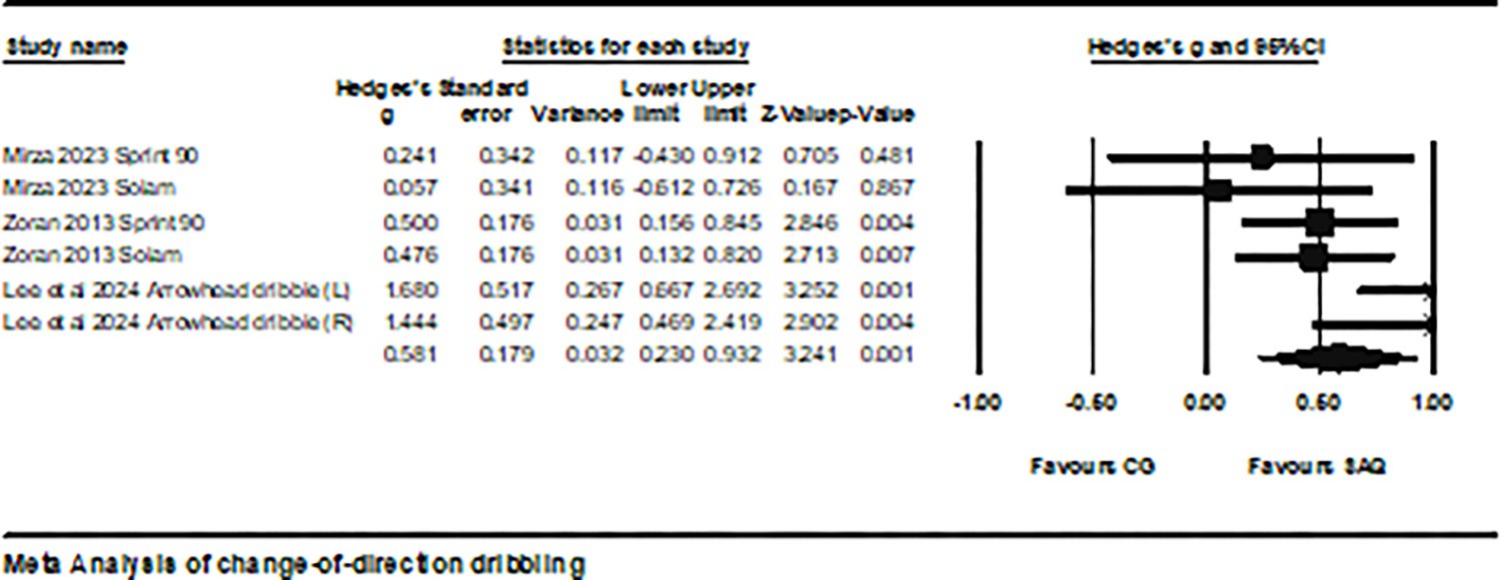
The forest plot illustrates the changes in change-of-direction dribbling performance between athletes participating in SAQ training and the control group. The plot shows effect sizes (Hedges’ g) along with their 95% confidence intervals (CI). The area of each square indicates the relative weight of the study.

Three studies involving data on change-of-direction dribbling performance were included, comprising six experimental groups and six control groups (total n = 184). The impact of SAQ on change-of-direction dribbling performance in soccer players approached a moderate effect size (ES = 0.58; 95% CI = 0.23–0.93; P = 0.01; *I*^2^ = 54.8%; Egger’s test p = 0.32; Fig 8). The weight of each study ranged from 8.89% to 25.66% in the analysis.

### Adverse effects

The analysis revealed that none of the studies reported negative outcomes related to SAQ training, including discomfort, pain, fatigue, injuries, or other health problems.

## Discussion

This study explored the impact of SAQ training on soccer player performance. The final analysis incorporated 9 studies that met the selection criteria. The findings revealed that SAQ training resulted in small to moderate enhancements (ES = 0.35 to 0.72) in sprint speed, COD, change-of-direction dribbling and both horizontal and vertical power in soccer players when compared to control groups. However, the improvement in flexibility was not significant compared to the control group. The heterogeneity of the aforementioned results ranged mostly from low to moderate (*I*^2^ = 0.0–54.8%), with sprint speed showing relatively higher heterogeneity (*I*^2^ = 75.6%). The study findings supported the research on the improvements in sprint speed, COD, power, and change-of-direction dribbling with SAQ intervention. While this study did not support the findings of two studies [14,37] on the 20-meter sprint, there were discrepancies in the significance reports compared to two other studies [15,21].

### The effect of SAQ on sprint speed

Short accelerations and linear sprints are considered crucial movements in soccer matches as they often precede goals and other decisive actions [39]. Our meta-analysis revealed that SAQ training can enhance the performance of soccer players in short-distance acceleration and sprinting (ES = 0.72) [11,14,15,20–22,36], particularly in 10-meter sprints (ES = 1.01) [14,21,36]. Although two studies reported significant improvements in the 25m [20] and 30m sprints [21], respectively, due to the limited number of studies, they were not included in the meta-analysis. Previous studies have found that optimization of lower body explosiveness is considered crucial for enhancing sprinting [19,40], and this optimization depends not only on muscular strength but also involves improved coordination between the nervous system and muscles [41]. SAQ exercises increase muscle strength, power, speed, and agility by altering or enhancing neural drive. Neural drive involves the generation and transmission of action potentials. Training contributes to enhancing neural drive by increasing the rate and quantity of action potential generation and transmission. Neurophysiological changes accompanying speed are associated with agility and speed training revolving around the stretch-shortening cycle (SSC) [42]. This muscle action can generate more efficient movements and help optimize the relative force produced by each recruited motor unit, thereby enhancing strength (jumping higher, sprinting faster) [43]. The results of this study also support the structural and neural adaptations occurring through SAQ training, thereby improving SSC function. Furthermore, the results of the meta-analysis revealed varying effects of SAQ on different sprint distances, with 10 meters being the most effective, followed by 5 meters (ES = 0.81), and the least effect observed for 20 meters (ES = 0.45). Regarding the effectiveness of 20-meter sprints, we noted that some studies reported significantly larger effects in samples aged 17 and above [15,21], while the studies reporting no significant effects targeted prepubescent children [14]. This finding is not surprising, as different age groups exhibit distinct characteristics in terms of neurological development, muscularity, and strength [44]. Research indicates that 10-11-year-old children may primarily benefit from explosive activities (based on rapid stretch-shortening cycles), as heightened neuromuscular adaptations contribute to their advantage in short-distance sprints (5m-15m) [36]. However, as maturity brings muscle and strength growth, this efficient neuromuscular adaptation may afford them an advantage at initiation [5], with differences of 30–50 centimeters (0.04–0.06 seconds/20 meters) potentially playing a decisive role in one-on-one contests [45].

### The effect of SAQ on agility

Agility is generally described as the capacity to swiftly alter speed and direction of movement in reaction to external cues [46]. These abilities are considered crucial in team sports, including soccer, as they involve essential adaptive skills [47,48]. This meta-analysis found that SAQ can help soccer players enhance their change of direction ability (ES = 0.35). Previous research indicates that decisive factors related to agility include cognitive (i.e., perceptual, decision-making) and physical (i.e., fitness, anthropometric indicators, technical skills, etc.) abilities [5]. Due to the low correlation between football-specific reactive agility (RAG) and change of direction speed (CODS) [49], it is imperative to clearly differentiate between CODS and reactive agility (RAG), especially in cognitive (i.e., perception, decision-making) and RAG testing [50]. Nonetheless, most of the studies included in this meta-analysis predominantly concentrated on CODS. While two studies reported significant effects of SAQ on reactive agility in football players [21,36], they did not meet the previously established criteria (more than two studies per analysis) for inclusion in the meta-analysis. Therefore, further research may be needed to investigate the impact of SAQ on reactive agility in soccer players. Lastly, it is noteworthy that one study included in this review reported a significant improvement in cognitive aspects for pre-adolescent football players following four weeks of SAQ training, three times per week, particularly in inhibitory control tasks (p = 0.029; ES = 1.10) [22]. The evaluation utilized two computer-based tasks: one measuring inhibitory control (Flanker task) and the other assessing perceptual speed (visual search task). This finding further illustrates the potential neural mechanisms underlying the relationship between exercise and certain forms of cognitive engagement and cognition [51–53], with SAQ training potentially being an effective means. However, due to the distinct characteristics of growth and development across different age groups [54], and the current lack of research pertaining to cognitive improvement outcomes across different age groups in relation to SAQ training, further studies are needed to explore the effects at various age stages.

### The effect of SAQ on power

In soccer matches, athletes need to perform various activities such as jumping, tackling, kicking, turning, and sprinting, the successful execution of which depends on the athletes’ maximal strength and rate of strength development [20]. Meta-analysis shows that SAQ training has a significant impact on the vertical and horizontal power of soccer players compared to control groups (ES = 0.67) [15,20]. As mentioned earlier, the advantage of SAQ training lies in improving various adaptation mechanisms, such as enhancing the recruitment of motor units, improving muscle coordination, enhancing neural drive to the agonist muscles, and enhancing the utilization of SSC rather than maximal force. This also explains why the study by Mario et al. (2011) [38] did not observe improvements in maximal strength following SAQ training. Furthermore, a substantial body of research has documented a strong correlation between lower limb power, agility, sprint speed, and vertical and horizontal jumping performance in elite soccer players [55–59]. Therefore, SAQ, as a continuum, may be a preferable choice for sports that require predominance in sprinting, agility, and power.

### The effect of SAQ on flexibility

Although previous research has indicated the importance of optimal flexibility levels in enhancing speed [60], and increasing flexibility in soccer players can reduce the risk of muscle injury [61], our meta-analysis found no evidence to suggest that SAQ training improves flexibility in soccer players (ES = 0.113), or that SAQ provides an advantage over conventional training in terms of flexibility. Although one study on female participants reported improvement with SAQ intervention, there were no significant differences compared to the control group, possibly due to females being inherently more flexible than males and thus showing easier improvement [62].

### The effect of SAQ on dribbling ability

The movement tasks and patterns in each stage of a soccer match involve players initiating or changing movements in different directions, whether with or without the ball [46,63,64]. The ability to swiftly dribble past opponents and penetrate into the opponent’s territory has long been regarded as a hallmark of genius [6], providing tactical advantages to one’s team [7,65]. Therefore, the assessment and monitoring of soccer players’ agility in executing specific tasks during rapid dribbling movements are deemed crucial [66]. Previous research has indicated that dribbling agility is associated not only with changes in sprinting ability, agility without the ball, and dynamic balance but also with physiological maturity and skill proficiency [67]. Similar to our observations, one study reported small effect sizes (Age < 12 years) [14], while two studies reported moderate to large effect sizes (age > 17 years) [21,36]. However, it is regrettable that there were no results regarding dynamic balance in any of the included studies. Indeed, change of direction (COD) requires good dynamic balance [68], and proficient balance can help reduce the risk of sports injuries during accelerating and decelerating processes [69,70]. While we found that studies in other sports disciplines have focused on the significant impact of SAQ on balance [71], it is necessary to further investigate the effects of SAQ on the balance of soccer players due to the distinct characteristics of different sports.

## Limitations

The systematic review has several notable limitations that must be reported. Firstly, the study only included research on soccer players, thereby precluding an assessment of the effects of SAQ on athletes in other sports. Secondly, due to the limited number of studies, meta-analyses could not be conducted to evaluate the impact of SAQ on outcomes such as aerobic endurance, anaerobic endurance [20], dribbling speed [21] and cognitive performance [22]. In addition, the observed effectiveness of SAQ training might vary across different age groups due to developmental differences in neuromuscular adaptability. Younger players (e.g., under 12 years) may exhibit greater improvements as their motor coordination and cognitive processing systems are highly plastic during early development [14,22]. However, the absence of studies including participants aged 13–17 in this review limits our understanding of SAQ’s impact during adolescence—a critical period for physical and skill development. Future research focusing on this age group could provide valuable insights into the age-specific applications of SAQ training. Moreover, this review included only two studies on female soccer players, which restricts our insight into the overall effectiveness of SAQ training for improving soccer performance. Thirdly, including results from studies with more than two articles in meta-analyses could strengthen the results; however, due to the limited number of articles, additional analyses on SAQ frequency, duration, total sessions, and weekly training time for different outcomes could not be performed. Therefore, specific recommendations for optimal training variables of SAQ to enhance soccer player performance in cognition, aerobic endurance, and anaerobic endurance cannot be provided. Finally, the publication search was restricted to studies written in English, potentially limiting the representativeness of the research findings.

## Conclusions

The results of this study demonstrate that SAQ training can effectively enhance the performance of adolescent soccer players. SAQ showed significant improvements in sprint speed, agility, horizontal and vertical power, as well as dribbling speed. However, no significant impact on flexibility performance was observed. However, to fully understand how SAQ training impacts cognition, balance, and various athletic skills, and to better assess its effect on match performance, additional high-quality research across a broader spectrum of sports is needed.

### Practical application

The conclusions of this review hold practical significance for soccer coaches, trainers, and athletes. SAQ continuum can serve as a training strategy to enhance the short-distance sprinting, dribbling, and agility, as well as explosiveness of soccer players. Additionally, an advantage of SAQ training is that its effectiveness remains nearly consistent regardless of the presence of specialized SAQ equipment, making it cost-effective and easily integrable into regular training programs [15,20]. However, further well-designed studies are needed to determine the optimal protocols and analyze the interaction between different training variables to benefit a wider population.

## Supporting information

S1 Table Detailed search strategy. (DOCX)

S2 Table Date used for meta-analysis. (DOCX)

S3 Table PRISMA 2020 checklist. (DOCX)

S4 Table List of all studies identified in the literature search. (XLSX)

S5 Table All data extracted from primary studies (for systematic reviews and meta-analyses). (XLSX)

S6 Table Risk of bias assessment and reasons for exclusion. (XLSX)
