# Supplementary material for: Effects of speed, agility, and quickness (SAQ) training on soccer player performance—a systematic review and meta-analysis
Source: PLoS One. 2025 Feb 21;20(2):e0316846. doi: 10.1371/journal.pone.0316846 (PMC11845227; doi:10.1371/journal.pone.0316846)
Supplement: S1 Table — (DOCX) [file pone.0316846.s001.docx]

**Table S1. Detailed search strategy.**

**Searched On March 25, 2024**

| **Databases** | **search strategy** | **Results** |
| --- | --- | --- |
| PubMed | (((SAQ training) OR (speed, agility, quickness training)) AND ((((((speed) OR (flexibility)) OR (agility)) OR (Athlete performance)) OR (Sports performance)) OR (physical performance))) AND ((((Soccer player*) OR (football player*)) OR (soccer athlete*)) OR (football athlete*)) | 11 |
| Web of Science Core Collection | “SAQ training” OR “Speed, Agility, and Quickness training” (All Fields) and "speed" OR "flexibility" OR "agility" OR "Athlete performance" OR "Sports performance" OR "physical performance" (All Fields) and "Soccer player*" OR "football player*" OR "soccer athlete*" OR "football athlete*" (All Fields) | 8 |
| SPORTDicus | ( "SAQ training" or "speed, agility, quickness training*" ) AND ( "speed" OR "flexibility" OR "agility" OR "Athlete performance" OR "Sports performance" OR "physical performance" ) AND ( "Soccer player*" OR "football player*" OR "soccer athlete*" OR "football athlete*" ) | 114 |
| Scopus | ( TITLE-ABS-KEY ( "SAQ training" OR "speed, agility, quickness training" ) AND TITLE-ABS-KEY ( "speed" OR "flexibility" OR "agility" OR "Athlete performance" OR "Sports performance" OR "physical performance" ) AND TITLE-ABS-KEY ( "Soccer player*" OR "football player*" OR "soccer athlete*" OR "football athlete*" ) ) | 12 |
| Total |  | 145 |
