# Supplementary material for: Effects of speed, agility, and quickness (SAQ) training on soccer player performance—a systematic review and meta-analysis
Source: PLoS One. 2025 Feb 21;20(2):e0316846. doi: 10.1371/journal.pone.0316846 (PMC11845227; doi:10.1371/journal.pone.0316846)
Supplement: S2 Table — (DOCX) [file pone.0316846.s002.docx]

| Sprint Data Collection | | | | | | | | | | | |  |  |  |
| --- | --- | --- | --- | --- | --- | --- | --- | --- | --- | --- | --- | --- | --- | --- |
| Sprint | | | | | | | | | | | |  |  |  |
|  | Study | EG/pre-mean | EG/pre-SD | EG/post-mean | EG/post-SD | EG=sample | CG/pre-mean | CG/pre-SD | CG/post-mean | CG/post-SD | CG=sample | week | F | frequency |
| 5m | Mirza et al.(5m) | 1.61 | 0.2 | 1.45 | 0.13 | 18 | 1.31 | 0.08 | 1.42 | 0.09 | 15 | 4 | 2 | 8 |
| 10m | Mirza et al.(10m) | 2.6 | 0.26 | 2.46 | 0.15 | 18 | 2.25 | 0.11 | 2.36 | 0.12 | 15 | 4 | 2 | 8 |
| 20m | Mirza et al.(20m) | 4.15 | 0.31 | 4.26 | 0.3 | 18 | 4.02 | 0.18 | 4.12 | 0.16 | 15 | 4 | 2 | 8 |
| 5m | Athos et al.(5m) 2022 | 1.27 | 0.08 | 1.22 | 0.09 | 11 | 1.28 | 0.05 | 1.24 | 0.06 | 10 | 4 | 3 | 12 |
| 20m | Athos et al.(20) 2022 | 3.96 | 0.29 | 3.9 | 0.28 | 11 | 4.07 | 0.19 | 4.04 | 0.21 | 10 | 4 | 3 | 12 |
| 5m | Zoran et al.(5m) 2014 | 1.42 | 0.16 | 1.4 | 0.16 | 66 | 1.44 | 0.08 | 1.46 | 0.08 | 66 | 12 | 4 | 48 |
| 10m | Zoran et al.(10m) 2014 | 2.14 | 0.16 | 2.07 | 0.08 | 66 | 2.17 | 0.16 | 2.21 | 0.16 | 66 | 12 | 4 | 48 |
| 20m | Zoran et al.(20m) 2014 | 3.35 | 0.16 | 3.34 | 0.16 | 66 | 3.37 | 0.16 | 3.37 | 0.16 | 66 | 12 | 4 | 48 |
| 25m | [Remco et al.(25m)EG1](https://pubmed.ncbi.nlm.nih.gov/?term=Polman+R&cauthor_id=14998097) | 4.31 | 0.09 | 4.14 | 0.07 | 12 | 4.33 | 0.12 | 4.3 | 0.21 | 12 | 12 | 2 | 24 |
| 25m | [Remco et al.(25m)EG2](https://pubmed.ncbi.nlm.nih.gov/?term=Polman+R&cauthor_id=14998097) | 4.33 | 0.12 | 4.12 | 0.13 | 12 | 4.33 | 0.12 | 4.3 | 0.21 | 12 | 12 | 2 | 24 |
| 5m | Athos et al.(5m) 2016 | 1.53 | 0.08 | 1.5 | 0.08 | 20 | 1.49 | 0.07 | 1.5 | 0.09 | 19 | 12 | 2 | 24 |
| 20m | Athos et al.(20m) 2016 | 4.27 | 0.24 | 4.16 | 0.26 | 20 | 4.11 | 0.22 | 4.05 | 0.23 | 19 | 12 | 2 | 24 |
| 5m | Young et al.(5m) | 1.45 | 0.08 | 1.35 | 0.05 | 9 | 1.41 | 0.08 | 1.38 | 0.07 | 10 | 8 | 3 | 24 |
| 10m | Young et al.(10m) | 2.3 | 0.06 | 2.14 | 0.09 | 9 | 2.3 | 0.09 | 2.21 | 0.04 | 10 | 8 | 3 | 24 |
| 20m | Young et al.(20m) | 3.74 | 0.14 | 3.57 | 0.12 | 9 | 3.67 | 0.2 | 3.74 | 0.17 | 10 | 8 | 3 | 24 |
| 30m | Young et al.(30m) | 5.73 | 0.23 | 5.49 | 0.1 | 9 | 5.22 | 0.13 | 5.24 | 0.29 | 10 | 8 | 3 | 24 |
| 20m | Umair et al.（20m) | 3.52 | 0.13 | 3.19 | 0.19 | 33 | 3.46 | 0.2 | 3.37 | 0.25 | 33 | 6 | 3 | 18 |

| Agility | | | | | | | | | | |  |  |  |  |
| --- | --- | --- | --- | --- | --- | --- | --- | --- | --- | --- | --- | --- | --- | --- |
| Study | EG/pre-mean | EG/pre-SD | EG/post-mean | EG/post-SD | EG=sample | CG/pre-mean | CG/pre-SD | CG/post-mean | CG/post-SD | CG=sample | Week | F | 次数 | Time |
| Mirza et al.(505L) | 3.06 | 0.23 | 3.07 | 0.21 | 18 | 2.9 | 0.13 | 2.83 | 0.09 | 15 | 4 | 2 | 8 | 20 |
| Mirza et al.505R) | 3.03 | 0.24 | 3 | 0.24 | 18 | 2.87 | 0.1 | 2.88 | 0.13 | 15 | 4 | 2 | 8 | 20 |
| Mirza et al.(4*5) | 7.54 | 0.45 | 7.38 | 0.51 | 18 | 7.19 | 0.33 | 6.97 | 0.34 | 15 | 4 | 2 | 8 | 20 |
| Mirza et al.(CODS90) | 8.74 | 0.95 | 7.81 | 0.76 | 18 | 8.03 | 0.44 | 7.24 | 0.48 | 15 | 4 | 2 | 8 | 20 |
| Athos et al.(CODS90) | 8.03 | 0.57 | 7.97 | 0.83 | 11 | 8.13 | 0.64 | 8.35 | 0.87 | 10 | 4 | 3 | 12 | 120 |
| Zoran et al.(4*5) | 5.93 | 0.38 | 5.86 | 0.39 | 66 | 6.04 | 0.35 | 6.07 | 0.34 | 66 | 12 | 4 | 48 | 120 |
| Zoran et al.(CODS90) | 7.83 | 0.51 | 7.67 | 0.48 | 66 | 7.72 | 0.63 | 7.75 | 0.65 | 66 | 12 | 4 | 48 | 120 |
| Zoran et al.(ST) | 7.83 | 0.74 | 7.77 | 0.76 | 66 | 7.85 | 1.06 | 7.95 | 1.13 | 66 | 12 | 4 | 48 | 120 |
| Zoran et al.(CODS180) | 7.4 | 0.33 | 7.29 | 0.35 | 66 | 7.46 | 0.35 | 7.49 | 0.36 | 66 | 12 | 4 | 48 | 120 |
| [Remco et al.EG1(180T)](https://pubmed.ncbi.nlm.nih.gov/?term=Polman+R&cauthor_id=14998097) | 2.76 | 0.18 | 2.63 | 0.14 | 12 | 2.75 | 0.19 | 2.7 | 0.18 | 12 | 12 | 2 | 24 | 60 |
| [Remco et al.EG2(180T)](https://pubmed.ncbi.nlm.nih.gov/?term=Polman+R&cauthor_id=14998097) | 2.74 | 0.09 | 2.62 | 0.13 | 12 | 2.75 | 0.19 | 2.7 | 0.18 | 12 | 12 | 2 | 24 | 60 |
| Athos et al.(ILL-T) | 13.35 | 0.66 | 13.4 | 0.52 | 20 | 13.1 | 0.69 | 13.08 | 0.68 | 19 | 12 | 2 | 24 | 30 |
| Young et al.(A-L) | 9.41 | 0.24 | 9.29 | 0.28 | 9 | 9.09 | 0.29 | 9.35 | 0.38 | 10 | 8 | 3 | 24 | 40 |
| Young et al.(A-R) | 9.32 | 0.27 | 9.1 | 0.27 | 9 | 9.12 | 0.21 | 9.27 | 0.32 | 10 | 8 | 3 | 24 | 40 |
| Young et al.(SEMO T) | 12.16 | 0.24 | 11.52 | 0.36 | 9 | 11.53 | 0.48 | 12.09 | 0.56 | 10 | 8 | 3 | 24 | 40 |
| Umair et al.(Iion-t) | 16.41 | 0.13 | 15.95 | 0.12 | 33 | 16.43 | 0.51 | 16.25 | 0.53 | 33 | 6 | 3 | 18 | 60 |

| Flexibility | | | | | | | | | | |
| --- | --- | --- | --- | --- | --- | --- | --- | --- | --- | --- |
|  | Sitting forward bend | | | | | | | | | |
| Study | EG/pre-mean | EG/pre-SD | EG/post-mean | EG/post-SD | EG=sample | CG/pre-mean | CG/pre-SD | CG/post-mean | CG/post-SD | CG=sample |
| Remco 2004 EG1 | 9.9 | 4.03 | 11.5 | 4.38 | 12 | 7.3 | 3.31 | 8.3 | 2.77 | 12 |
| Remco 2004 EG2 | 7.8 | 3.02 | 8.9 | 2.39 | 12 | 7.3 | 3.31 | 8.3 | 2.77 | 12 |
| Zoran 2014 | 12.42 | 5.61 | 12.8 | 5.61 | 66 | 14 | 3.49 | 13.82 | 3.49 | 66 |

| Dribbling agility | | | | | | | | | | |
| --- | --- | --- | --- | --- | --- | --- | --- | --- | --- | --- |
| Study | EG/pre-mean | EG/pre-SD | EG/post-mean | EG/post-SD | EG=sample | CG/pre-mean | CG/pre-SD | CG/post-mean | CG/post-SD | CG=sample |
| Mirza 2023 Sprint 90 | 14.26 | 1.88 | 12.99 | 1.77 | 18 | 12.45 | 1.18 | 11.55 | 1.07 | 15 |
| Mirza 2023 Solam | 16.11 | 1.53 | 16.18 | 1.6 | 18 | 14.01 | 1.39 | 14.17 | 1.47 | 15 |
| Zoran 2013 Sprint 90 | 9.92 | 0.6 | 9.67 | 0.58 | 66 | 9.85 | 0.64 | 9.91 | 0.65 | 66 |
| Zoran 2013 Solam | 10.93 | 1.11 | 10.67 | 1.06 | 66 | 10.95 | 1.21 | 11.24 | 1.23 | 66 |
| Young 2024 Arrowhead dribble (L) | 10.96 | 0.28 | 10.7 | 0.34 | 9 | 11.14 | 0.58 | 11.64 | 0.5 | 10 |
| Young 2024 Arrowhead dribble (R) | 10.99 | 0.42 | 10.74 | 0.49 | 9 | 10.68 | 0.44 | 11.22 | 0.55 | 10 |

| Explosive | | | | | | | | | | |
| --- | --- | --- | --- | --- | --- | --- | --- | --- | --- | --- |
| Study | EG/pre-mean | EG/pre-SD | EG/post-mean | EG/post-SD | EG=sample | CG/pre-mean | CG/pre-SD | CG/post-mean | CG/post-SD | CG=sample |
| Umair 2021 | 42.85 | 3.19 | 46.68 | 3.88 | 33 | 43.37 | 8.56 | 44.76 | 8.06 | 33 |
| Remco 2004 EG1 VJ | 39.9 | 3.32 | 46.6 | 3.23 | 12 | 39.3 | 5.67 | 41.2 | 4.39 | 12 |
| Remco 2004 EG2VJ | 38.8 | 4.11 | 46.6 | 4.81 | 12 | 39.3 | 5.67 | 41.2 | 4.39 | 12 |
| Remco 2004 EG1 HJ | 183 | 14 | 198 | 16 | 12 | 184 | 19 | 187 | 14 | 12 |
| Remco 2004 EG2 HJ | 181 | 10 | 194 | 8 | 12 | 184 | 19 | 187 | 14 | 12 |
